# Growth parameter estimation and model simulation for three industrially relevant microalgae: *Picochlorum, Nannochloropsis*, and *Neochloris*

**DOI:** 10.1002/bit.28052

**Published:** 2022-02-19

**Authors:** Robin Barten, Rocca Chin‐On, Jeroen de Vree, Ellen van Beersum, Rene H. Wijffels, Maria J. Barbosa, Marcel Janssen

**Affiliations:** ^1^ Bioprocess Engineering & AlgaePARC Wageningen University and Research Wageningen The Netherlands; ^2^ Water‐ en Energiebedrijf Bonaire Kralendijk Bonaire; ^3^ Biosciences and Aquaculture Nord University Bodø Norway

**Keywords:** growth model, microalgae, parameter estimation, photobioreactor, *Picochlorum*

## Abstract

Multiple models have been developed in the field to simulate growth and product accumulation of microalgal cultures. These models heavily depend on the accurate estimation of growth parameters. In this paper growth parameters are presented for three industrially relevant microalgae species: *Nannochloropsis* sp., *Neochloris oleoabundans*, and *Picochlorum* sp. *(BPE23)*. Dedicated growth experiments were done in photobioreactors to determine the maximal biomass yield on light and maintenance rate, while oxygen evolution experiments were performed to estimate the maximal specific growth rate. *Picochlorum* sp. exhibited the highest specific growth rate of 4.98 ± 0.24 day^−1^ and the lowest specific maintenance rate of 0.079 day^−1^, whereas *N. oleoabundans* showed the highest biomass yield on light of 1.78 g_x_·mol_ph_^−1^. The measured growth parameters were used in a simple kinetic growth model for verification. When simulating growth under light conditions as found at Bonaire (12 °N, 68° W), *Picochlorum* sp. displayed the highest areal biomass productivity of 32.2 g.m^−2^·day^−1^ and photosynthetic efficiency of 2.8%. The presented growth parameters show to be accurate compared to experimental data and can be used for model calibration by scientists and industrial communities in the field.

## Graphical abstract

The growth parameters: maximal biomass yield on light, maintenance rate, and maximal specific growth rate were estimated through dedicated laboratory experiments for three industrially relevant microalgal species: *Picochlorum, Nannochloropsis*, and *Neochloris*. The estimated growth parameters were then used in a growth model to simulate biomass productivity at an industrial scale. Other scientists can use the estimated growth parameters as input data in their models.

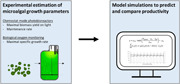

## 1 INTRODUCTION

Microalgae are a promising sustainable platform to produce proteins, lipids, carbohydrates and pigments. However, commercialization is still limited by high production costs (Draaisma et al., 2013; Oostlander et al., 2020; Wijffels & Barbosa, 2010). These costs need to be significantly reduced to create a profitable business model. The production of microalgae commonly takes place in cultivation systems, such as raceway ponds and photobioreactors (Vree et al., 2015). Many studies attempt at optimizing the microalgae production process through a variety of measures. Verification of the impact of these measures and optimization of the production process is commonly done through mathematical simulations before upscaling to pilot and production scale (Darvehei et al., 2018).

Many mathematical models have been developed, and are still being developed, to predict biomass productivity in photobioreactors (Blanken et al., 2016; Oostlander et al., 2020; Quinn et al., 2011; Ruiz et al., 2016). Applications of these models are for example: predictions on the impact of different photobioreactor designs, predictions on different operation modes, and predictions of the effect of different climatological conditions (Darvehei et al., 2018; Lee et al., 2015). One thing that all these models have in common is their dependency on accurate input parameters. Examples of important biological parameters for modeling microalgal growth are the maximal specific growth rate, the maximal biomass yield on light (i.e., intrinsic photosynthetic efficiency), and the specific maintenance rate of the microalgal species. However, to determine these model parameters for microalgae accurately and systematically is labor intensive. Such growth parameters are seldomly determined for microalgal species. Not only due to the required time, but also due to the knowledge gap regarding simple methodology to estimate these parameters. The efficiency and rate at which microalgae convert light into biomass differ significantly between species (Kliphuis et al., 2012; León‐Saiki et al., 2018; Sforza et al., 2015). Considering this, growth parameters need to be determined anew for each species. As a result, there is a lack of reliable estimates of growth parameters to make accurate model predictions to simulate the impact of innovative methods on upscaling of algal cultivation (Darvehei et al., 2018).

The present study provides microalgal growth model parameters for three microalgae species that are of industrial interest: *Picochlorum* sp., *Neochloris oleoabundans*, and *Nannochloropsis* sp. (Barten et al., 2020; Li et al., 2008; Ma et al., 2016). The maximal photosynthetic efficiency (i.e., biomass yield on light), maintenance rate, and maximal specific growth rate were determined for each microalga in dedicated laboratory scale experiments. Flat‐panel photobioreactors and a biological oxygen monitor device were used as reliable and quantifiable systems to measure these parameters. An already existing and validated kinetic growth model was then applied to simulate and compare microalgae productivity at an ideal low‐latitude location using the newly obtained growth parameters (Blanken et al., 2016). The model parameters can be used by others as input data in other mathematical models to simulate microalgal cultivation systems.

## 2 MATERIAL AND METHODS

This study applied cell cultivation in flat‐panel photobioreactors and a biological oxygen monitor to determine growth parameters for three selected microalgae. These growth parameters then were used as input data in a microalgae growth model to assess and compare the productivity of the three microalgae species.

### 2.1 Cell cultivation

#### 2.1.1 Pre‐culture and growth media

*Picochlorum* sp. *(BPE23)* was isolated from a saltwater body of Bonaire (12 °N, 68° W) and pre‐cultivated in shake flasks in an orbital shaker incubator (Multitron, Infors HT) with a 12/12 h day/night cycle and an average light intensity of 100 μmolph m^−2^ s^−1^ (Barten et al., 2020). The temperature was 40°C during the day and 30°C during the night. The incubator's relative air humidity was set to 60% and enriched with 2% CO2. Cells were cultured in artificial seawater enriched with nutrients and trace elements. Salts, nutrients and trace elements were provided at the following concentrations (in g·L^−1^): NaCl, 24.5; MgCl_2_·6H_2_O, 9.80; Na_2_SO_4_, 3.20; CH_4_N_2_O (urea), 2.12; K_2_SO_4_, 0.85; CaCL_2_·2H_2_O, 0.80; KH_2_PO_4_, 0.23; Na_2_EDTA·2H_2_O, 0.105; Na_2_EDTA, 0.06; FeSO_4_·7H_2_O, 0.0396; MnCl_2_·2H_2_O, 1.71·10^−3^; ZnSO_4_·7H_2_O, 6.60·10^−4^; Na_2_Mo_4_·2H_2_O, 2.42·10^−4^; Co(NO_3_)_2_·6H_2_O, 7.00·10^−5^; NiSO_4_·6H_2_O, 2.63·10^−5^; CuSO_4_·5H_2_O, 2.40·10^−5^; K_2_CrO_4_, 1.94·10^−5^; Na_3_VO_4_, 1.84·10^−5^; H_2_SeO_3_, 1.29·10^−5^. HEPES (4.77 g·L^−1^) was added as a pH buffer to shake flask cultures. The medium pH was adjusted to 7.0 after which it was filter sterilized before use. Antifoam B (J.T. Baker; Avantor) was added at a concentration of 0.5 ml·L^−1^ from a 1% w/w% stock. Sodium bicarbonate (NaHCO₃)(0.168 g·L^−1^) was added during the inoculation to provide sufficient CO_2_ at the start of the cultivation.

*N. oleoabundans* UTEX1185 and *Nannochloropsis* sp. CCAP 211/78 pre‐cultures were cultivated in an orbital shaker incubator (Multitron, Infors HT), and illuminated with 50 μmol_ph_ m^−2^ s^−1^. Temperature was set to 25°C, and the headspace was enriched with 2% CO_2_. *N. oleoabundans* and *Nannochloropsis* sp. were cultivated on filtered natural seawater (Oosterschelde, The Netherlands) enriched with the following nutrients and trace elements (in g∙L^−1^): KH_2_PO_4_, 0.23; Na_2_EDTA, 0.21; Fe_2_SO_4_∙7H_2_O, 0.03; MnCl_2_∙2H_2_O, 1.62∙10^−3^; ZnSO_4_∙7H_2_O, 6.61∙10^−4^; Co(NO_3_)_2_∙6H_2_O, 6.98∙10^−4^; CuSO_4_∙5H_2_O, 2.50∙10^−4^; Na_2_MoO_4_∙2H_2_O, 2.66∙10^−3^. HEPES (4.77 g∙L^−1^) and Na_2_EDTA (1.86 g∙L^−1^) were added to the seawater as a pH buffer to Erlenmeyer cultures. The medium pH was adjusted to 7.5 followed by heat sterilization (20 min at 121°C). The nutrient and trace element solution was first autoclaved, and then filtrated through a sterile filter (0.20 µm). Sodium nitrate (2.13 g∙L^−1^) was used as nitrogen source to cultivate *Nannochloropsis* sp. CCAP 211/78, whereas urea (1.50 g∙L^−1^) was used for *N. oleoabundans*.

#### 2.1.2 Photobioreactor operation to estimate yield on light and maintenance rate

Microalgae were cultivated in sterilized flat panel photobioreactors (Algaemist; Technical Development Studio, WUR) with a 0.38 L working volume and a 14 mm optical depth (De Mooij et al., 2015). Flat panel photobioreactors were chosen for these experiment as these systems allow for the most accurate light calibration which is essential for accurate estimation of growth parameters. Photobioreactors were operated in chemostat mode at different dilution rates to determine the growth parameters: maximal yield of biomass on light ($Y_{\mathrm{xphm}}$) and specific maintenance rate (${µ}_{e}$). The photobioreactor was continuously illuminated (24/24 h) with warm white LED lamps (Bridgelux, BXRA W1200). Optimal growth temperatures were chosen for each microalgae species (Barten et al., 2020; de Vree, 2016). The CO_2_ level in the photobioreactor was non‐limiting, as controlled by the pH level through on‐demand CO_2_ supplementation. The outgoing light was measured with an external LI‐COR SA‐190 Quantum sensor (PAR‐range 400–700 nm). Photobioreactor harvest was collected in darkness at 4°C and weighed daily to monitor the photobioreactor dilution rate.

The photobioreactor was operated until a steady state was reached. The steady state was defined as followed: the variation on the measurements of optical density and dilution rate should remain <15% for at least three days. After reaching this steady state, measurements were performed daily for an additional seven days after which the average of the steady state was taken for data presentation.

#### 2.1.3 Biological oxygen monitor to estimate maximal photosynthetic rates

The maximal specific growth rate ($\mu_{\max}$) was indirectly estimated by monitoring the photosynthetic oxygen production rate with a liquid phase biological oxygen monitor (BOM) (Oxytherm^+^, Hansatech). An exponentially growing cell culture was diluted to a density of 0.1 to 0.4 g·L^−1^ in 2.4 ml volume and inserted into the measurement cuvette of the BOM. This low cell density was selected to minimize in‐culture cell shading. The medium was buffered at pH 7 with 20 mM HEPES and enriched with 50 mM sodium bicarbonate. Oxygen was stripped from the cell culture with nitrogen gas before closure of the cuvette. The microalgal culture was first exposed to darkness followed by increasing light levels while the oxygen concentration in the liquid was continuously recorded to generate a photosynthesis irradiance curve (PI curve). During this sequence the suspension was continuously mixed by a magnetic stirring bar and temperature was controlled at the optimal growth temperature (Table 1). Applied light levels are displayed in Supporting Information Appendix 6. The increase in dissolved oxygen over time was then used to calculate the specific growth rate.

**Table 1:** Experimental settings applied in the chemostat experiments to determine the maximal biomass yield on light and the maintenance rate

Species	*Nannochloropsis* sp.	*Neochloris oleoabundans*	*Picochlorum* sp.
Applied dilution rates (day^−1^)	0.1, 0.2, 0.4, 0.5, 0.6	0.4, 0.5, 0.6, 0.7, 0.8, 0.9, 1.2	0.18, 0.27, 0.31, 0.35, 0.48, 0.48, 0.57, 0.69, 0.90
Incident light intensity (μmol_ph_·m^‐2^·s^−1^)	100	100	100
Temperature (°C)	25	30	39
Air flow rate (vesselvolume·min^−1^)	2	2	1

### 2.2 Analysis

#### 2.2.1 Dry weight

The biomass concentration (in g·L^−1^) was measured in duplicate as dry weight. Empty Whatman glass microfiber filters (θ 55 mm, pore size 0.7 μm) were dried overnight at 95°C and placed in a desiccator for 2 h. Filters were then weighed and placed in a mild vacuum filtration setup. Culture containing 1–10 mg of microalgae biomass was diluted in 25 ml 0.5 M ammonium formate and filtered. The filter was washed twice with 25 ml 0.5 M ammonium formate to remove residual salts. The wet filter was dried overnight at 95°C, placed in a desiccator for 2 h, and weighed. Biomass concentration was calculated from the difference in filter weight before and after filtration, and the volume of the sample.

#### 2.2.2 Absorption cross‐section

The average dry‐weight specific optical cross section (m^2^·g^−1^) was measured with an UV‐VIS/double‐beam spectrophotometer (Shimadzu UV‐2600, light path: 2 mm), equipped with an integrating sphere module (ISR‐2600). Absorbance was measured from 400 to 700 nm with a step size of 1 nm.

### 2.3 Calculations

The μ: specific growth rate (day^−1^) was determined over a range of incident irradiance levels (Supporting Information Appendix 6) using the biological oxygen monitor and Equation (1). With $r_{o}$: the oxygen production rate (mol·L^−1^·day^−1^), $x_{\mathrm{ox}}$: the ratio of oxygen per biomass produced (mol·mol^−1^), and $C_{\mathrm{xcuvette}}$: biomass concentration in the culture chamber (mol·L^−1^). A molecular weight for microalgal biomass of 24 g· mol^−1^ was adopted from the literature (Blanken et al., 2016). The ratio in mole of oxygen per mole of biomass ($x_{\mathrm{ox}}$) was 1.11 for urea, and 1.44 for nitrate, as nitrogen source (Kliphuis et al., 2012). $\mu_{\max}$ was found at the light intensity where *μ* was largest.

(1) $$\mu =\frac{r_{o}}{x_{\mathrm{ox}}∙C_{\mathrm{xcuvette}}}$$

The maximal biomass yield on light and the maintenance rate were determined by chemostat experiments under light limited conditions. First the specific growth rate, μ (day^−1^), was calculated through the photobioreactor dilution rate (day^−1^) using Equation (2). With: $M_{h}$: harvest mass, (g·day^−1^), $V_{r}$: photobioreactor volume (0.38 L), and ρ: culture medium density (1030 g·L^−1^).

(2) $$\mu =D=\frac{M_{h}}{V_{r}\rho }$$

Through Equation (3), which is based on Pirt's law, a linear regression was made for the photobioreactor experiments by varying the reactor dilution rate. With as a result *μ* and *q_ph_*: the specific photon consumption rate (Kliphuis et al., 2012; S. Pirt, 1965). The equation was built on the assumption that all absorbed photons are used at maximal efficiency because of the application of light limitation and absence of photo saturation. This assumption is only valid under low light conditions as in the performed experiments, and evidenced by a linear correlation between μ and $q_{\mathrm{ph}}$ (Jassby & Platt, 1976; S. J. Pirt, 1982). The slope in this regression is equal to $\frac{1}{Y_{\mathrm{xphm}}}$ and the intercept with the *y*‐axis $m_{s}=\frac{\mu_{e}}{Y_{\mathrm{xphm}}}$.

(3) $$q_{ph}=\frac{\mu }{Y_{\mathrm{xphm}}}-\frac{\mu_{e}}{Y_{\mathrm{xphm}}}=\frac{I_{\mathrm{phin}}-I_{\mathrm{phout}}}{C_{x}d}$$

### 2.4 Model simulations

The obtained biological parameters (i.e., $Y_{\mathrm{xphm}},\mu_{e},\text{an}d\;\mu_{\max}$) for *Picochlorum* sp., *Nannochloropsis* sp., and *N. oleoabundans* were used in an existing kinetic model for microalgal growth under light limited conditions (Blanken et al., 2016) to simulate and compare their potential biomass productivities in a flat panel photobioreactor. This model was validated and proved to be accurate for simulating microalgal growth on lab‐scale (Blanken et al., 2016; Tuantet et al., 2019). The model calculations are based on the Lambert‐Beer law, the photosynthesis model of Jassby and Platt, and the aerobic chemoheterotrophic growth model of Pirt, and were applied to calculate the biomass productivity in case of chemostat operation as an example to showcase the measured biological growth parameters (Chalker, 1980; Jassby & Platt, 1976). It must be noted that the obtained parameters can also be used in other microalgal growth models that may be more sophisticated or in which nonideal growth conditions are simulated.

In our simulations, it was assumed that the photobioreactor is located on the Caribbean island Bonaire (12° N, 68° W) as an example case. Irradiance data was obtained from Meteonorm 7.1, a global climate database, in which measured irradiance over recent decades at the nearby weather station on Curacao is used to generate hourly data for a typical year. Of this data, day 172 was considered (Supporting Information Appendix 2), which is equal to the summer solstice, that is, the longest day in the northern hemisphere. Based on the irradiance values, light intensities on Day 172 were simulated as a sinus function in which a maximum intensity of 1900 μmol_ph_·m^−2^·s^−1^ is reached at noon, sunrise occurs at 6:00 a.m. and sunset occurs at 6:00 p.m.

Using the simulated light intensity, the Lambert‐Beer law is applied in the model to compute the light gradient over the depth of a flat horizontal photobioreactor (Equation 4). The Lambert–Beer law is a simple and commonly used method to model light. For the purpose of verifying the estimated growth parameters in flat panel photobioreactor systems the applied method provides sufficient accuracy. In research where the aim is to provide more accurate growth estimations for complicated reactor designs or weather patterns, more refined light models may be required, which include variation in the direction of light and light scattering (e.g., ray tracing or Monte‐Carlo simulations).

The applied model takes into account the spectrum of the sun, the depth of the photobioreactor, the absorption cross‐section of the microalgae, and the biomass concentration of the culture.

(4) $$I_{ph}\left(z\right)=\sum_{\lambda =700}^{\lambda =400}I_{ph,\lambda }\left(0\right)\cdot e^{-a_{x,\lambda }\cdot C_{x}\cdot z}\cdot ∆\lambda$$

In which $I_{\mathrm{ph}}(z)$ is the light intensity at depth *z* in the cell culture (mol_ph_·m^−2^·s^−1^), $I_{\mathrm{ph},\lambda }\left(0\right)$ is the light intensity for each wavelength in the PAR‐region at the illuminated surface of the cell culture (mol_ph_·m^−2^·s^−1^), $a_{x,\lambda }$ is the wavelength‐dependent absorption cross‐section (m^2^. mol_x_^−1^), $C_{x}$ is the biomass concentration (mol_x_·m^−3^), z is the distance from the illuminated reactor surface to depth *z* in the culture (m), and λ is the wavelength of the light in the PAR‐region (nm).

In our simulations, it was assumed that the depth of the reactor is equal to 0.015 m. This is an arbitrary choice and lies within a normal range for flat panel photobioreactors. The optical depth of the reactor ultimately influences the biomass concentration and can be further optimized at a later stage. The volume of the reactor itself does not influence the model results. For the absorption cross‐sections $a_{x,\lambda }$ of the microalgae, values for wavelengths between 400 and 700 nm were obtained from experiments in this study and from literature for low light conditions (Supporting Information Appendix 3).

From the light gradient within the reactor, the model ultimately calculates the average specific growth rate (Equation 5), based on the photosynthesis model of Jassby and Platt and the aerobic chemoheterotrophic growth model of Pirt.

(5) $$\mu =\mu_{m}\cdot \tanh\left(\frac{Y_{\mathrm{xphm}}\cdot a_{x}∙I_{ph}}{\mu_{m}}\right)-\mu_{e}$$

In which μ is the average specific growth rate (s^−1^), $\mu_{m}$ is the maximum specific growth rate (s^−1^), $Y_{\mathrm{xphm}}$ is the maximum biomass yield on light (mol_x_·molph^−1^), and $\mu_{e}$ is the maintenance rate (s^−1^).

The areal biomass productivity and the biomass yield on light were calculated by the model for chemostat operation conditions where the dilution was set for the hours between sunrise and sunset. A period of 10 identical days was simulated, which was found to be sufficient to reach a pseudo steady‐state characterized by a repetitive cyclic pattern of the biomass concentration. For each of the microalgae, a dilution rate was chosen that results in the highest biomass productivity, namely 0.68 day^−1^ for *Picochlorum* sp., 0.47 day^−1^ for *N. oleoabundans*, and 0.44 day^−1^ for *Nannochloropsis* sp. (Supporting Information Appendix 4).

The changes in biomass concentration in the photobioreactor as well as the harvested biomass were computed for every minute of the simulated period, based on the following equations:

(6) $$\frac{d}{\mathrm{dt}}C_{x}(t)=(\mu (C_{x}(t),t)-D\left(t\right))∙\;C_{x}(t)$$

(7) $$\frac{d}{\mathrm{dt}}M_{x}(t)=D\left(t\right)∙\;C_{x}\left(t\right)$$

In which D is the dilution rate (s^−1^) and $M_{x}$ is the harvested biomass (mol·m^−3^).

Since the average specific growth rate μ itself is a function of the biomass concentration in the photobioreactor, a Runge–Kutta method was adopted to solve the differential equations for $C_{x}$ and $M_{x}$ over the simulated period of 10 days. The initial biomass concentrations were chosen to be 2.9 g·L^−1^ for *Picochlorum* sp., 3.6 g·L^−1^ for *N. oleoabundans*, and 3.6 g·L^−1^ for *Nannochloropsis* sp., which were found to be near the final steady‐state concentrations.

Ultimately, the areal biomass productivity and the observed biomass yield on light on the 10th day were considered. These were calculated using the following equations:

(8) $$r_{x,\mathrm{area}}=(C_{x}(t_{\mathrm{day}10})-C_{x}(t_{\mathrm{day}9}))+(M_{x}(t_{\mathrm{day}10})-M_{x}(t_{\mathrm{day}9}))∙\;l$$

(9) $$Y_{x/ph}=\frac{r_{x,\mathrm{area}}}{I_{ph}}$$

In which $r_{x,\mathrm{area}}$ is the areal biomass productivity (mol·m^−2^·day^−1^), l is the photobioreactor depth (m), $Y_{x/\mathrm{ph}}$ is the observed biomass yield on light (mol_x_·mol_ph_^−1^), and $I_{\mathrm{ph}}$ is the total available irradiance during the simulated day (mol_ph_). From the areal biomass productivity and the available irradiance, the photosynthetic efficiency was also determined as described in Supporting Information Appendix 5.

## 3 RESULTS AND DISCUSSION

### 3.1 Biomass yield on light and specific maintenance rate

**Figure 1:**
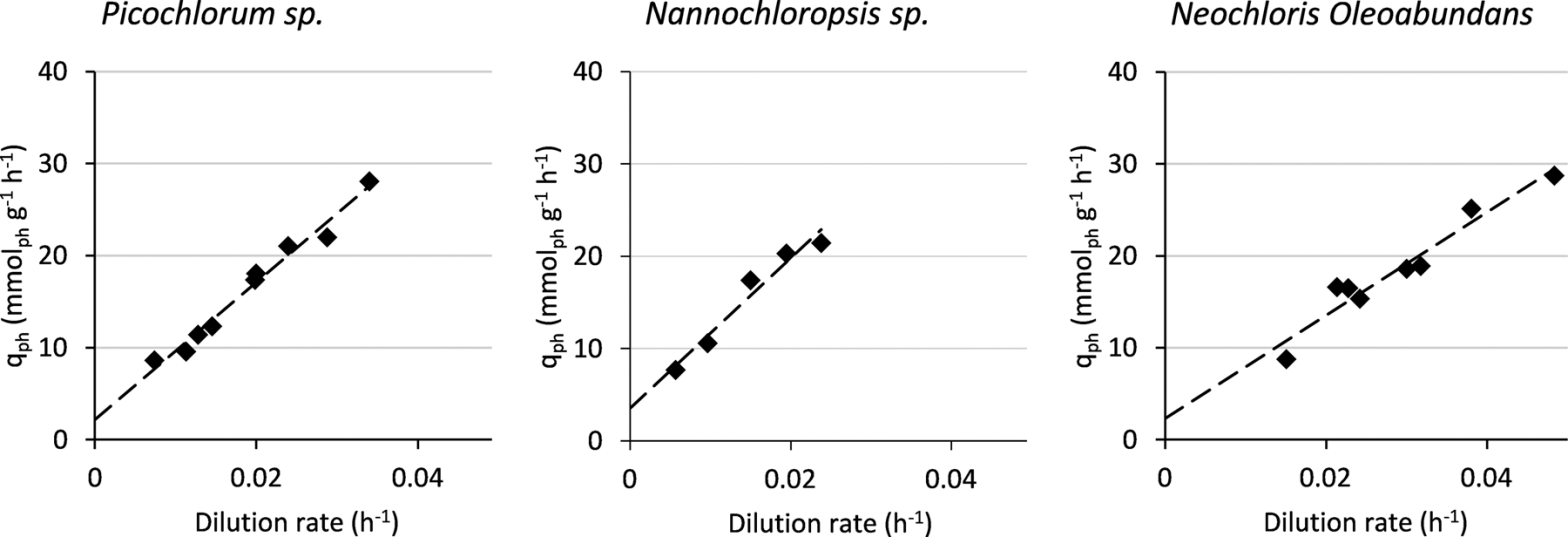
Specific photon consumption rate as a function of the photobioreactor dilution rate for three microalgae species, *Picochlorum* sp. (BPE23), *Neochloris oleoabundans*, and Nannochloropsis sp. Each data point is the average ± SD of 7 days of steady‐state growth. A linear regression was plotted to indicate the intercept with the *y*‐axis

The maximal biomass yield on light and specific maintenance rate were determined through light limited chemostat experiments for the microalgae *Picochlorum* sp. *(BPE23), Nannochloropsis* sp. and *N. oleoabundans*. Currently, the most accurate method for estimation of the maximal yield on light and the specific maintenance rate is based on the application of chemostat‐operated photobioreactors. In this situation, a steady state will be reached, allowing for measurements on a culture that does not change over time. This approach is essential for photoautotrophic growth due to the extra dimension of light. Changing biomass concentrations during phototrophic batch growth will cause changing substrate (i.e., light) levels due to in‐culture cell shading. In comparison, for heterotrophic growth, steady‐state conditions are of less importance as changing biomass concentrations will not directly affect substrate availability, and therefore, batch experiments are often used to determine the biomass yield on substrate. A low incident irradiance level of 100 μmolph m^−2^ s^−1^ was set to ensure that all light was used for growth without energy wastage through light dissipation in the photosynthetic complexes. The specific photon consumption: $q_{\mathrm{ph}}$ rate was plotted as a function of the reactor dilution rate, and thus as the specific growth rate (µ) (Equation 2) for each of the performed experiments (Figure 1). This resulted in a linear relation between the different runs. The linear relation observed for each of the microalgae confirms that light limited conditions were achieved, as light saturated cells would have resulted in a negative exponential relationship (Jassby & Platt, 1976; S. J. Pirt, 1982). Therefore, we conclude that almost all energy was utilized for growth and that energy dissipation as a result of photosystem oversaturation was minimal. A linear regression was made for each data set (Figure 1). The inverse of these regression lines' slopes represents the maximal biomass yield on light ($Y_{\text{xphm}}$). The specific maintenance rate (${µ}_{e}$) of the microalgae species was deducted from the point at which the regression line intercepts the y‐axis.

An overview of the obtained model parameters is presented in Table 2. The maximal biomass yield on light for *Picochlorum* sp. *(BPE23)* was determined to be 1.38 g_x_·mol_ph_^−1^. For *Nannochloropsis* sp. a lower yield on light of 1.23 g_x_·mol_ph_^−1^ was measured, while for *N. oleoabundans* a higher yield on light of 1.78 g_x_·mol_ph_^−1^ was measured. The maximal theoretical yield on light is 1.5 and 1.8 g_x_·mol_ph_^−1^ for growth on nitrate and urea, respectively (Zijffers et al., 2010). In practice, microalgal species show a yield on light ranging from 1.0 to 1.8 g_x_·mol_ph_^−1^, depending on species. The model organisms *Chlamydomonas reinhardtii* and *Chlorella sorokiniana* display a yield on light of 1.25 and 1.80 g_x_·mol_ph_^−1^, respectively (Kliphuis et al., 2012; Kliphuis et al., 2011), while *Dunaliella salina* and *Tetradesmus obliquus* present a yield on light of 1.00 and 1.15 g_x_·mol_ph_^−1^ (Fachet et al., 2014; León‐Saiki et al., 2018). A study done on *Nannochloropsis salina* shows a yield on light of 1.68 g_x_·mol_ph_^−1^, which is high compared with the yield on light of 1.23 g_x_·mol_ph_^−1^ that we found for *Nannochloropsis* sp. in our study (Sforza et al., 2015).

**Table 2:** Values for the maximal biomass yield on light ($Y_{\mathrm{xphm}}$) and maintenance rate ($\mu_{e}$), as found through photobioreactor experiments

Organism	$Y_{\mathrm{xphm}}$ (g_x_·mol_ph_^−1^)	$\mu_{e}$ (day^‐1^)
*Picochlorum* sp. (BPE23)	1.38	0.079
*Nannochloropsis* sp.	1.23	0.099
*Neochloris oleoabundans*	1.78	0.104

The maximal yield on light varies significantly between species, and even between strains. The cause for the variation is difficult to pinpoint and would require in‐depth study of each strains' energy metabolism. To a certain extent the yield on light is influenced by the biomass composition, which can cause a small error in the estimated growth parameters. Also the efficiency in energy transfer and carbon fixation can lead to differences in photosynthetic efficiency (Geider & Osborne, 1989). Another potential cause is the absorption of light by filtering pigments, which reduces the available photons for biomass formation (Mulders et al., 2014).

The specific maintenance rate (${µ}_{e}$) was estimated experimentally. *Picochlorum* sp. *(BPE23)* showed a specific maintenance rate of 0.079 day^−1^, whereas *Nannochloropsis* sp. and *N. oleoabundans* showed a specific maintenance rate of 0.099 and 0.104 day^−1^, respectively. This maintenance rate equals the rate at which microalgal biomass decreases when irradiated, and in darkness. To facilitate maintenance during dark periods, microalgae provide energy from reserves through respiration. The estimated specific maintenance rates in our study are average to low compared with values found in literature. Few microalgal species from the class of *Chlorophyceae* such as *Chlorella pyrenoidosa*, *Dunaliella tetriolecta*, and *Nannochloropsis atomus* exhibit specific maintenance rates of 0.08, 0.18, and 0.14 day^−1^, respectively (Geider & Osborne, 1989).

### 3.2 Maximal growth rate

**Figure 2:**
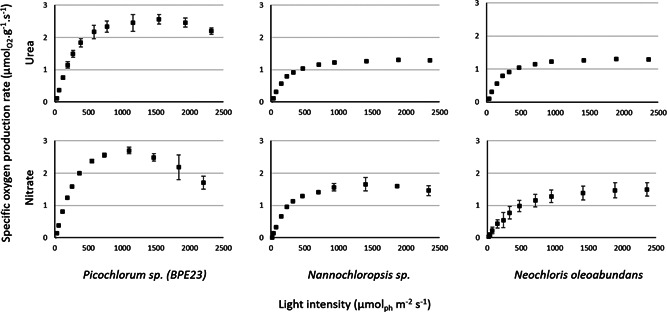
PI curves for *Picochlorum* sp. (BPE23), *N. oleoabundans*, and *Nannochloropsis* sp., grown with either urea or nitrate as nitrogen source. The *y*‐axis displays the specific oxygen production rate (μmol_O2_·g^−1^·s^−1^) at different light levels, measured by the biological oxygen monitor (BOM). Data represent the average ± SD of at least three biological replicates

The microalgal net specific oxygen production rate ($q_{o}$) was measured for *Picochlorum* sp. *(BPE23)*, *Nannochloropsis* sp., and *N. oleoabundans* when grown on urea and nitrate, using a range of light intensities (Figure 2). From the specific oxygen production rate we estimated the maximal specific growth rate (µ_*max*_) based on biomass stoichiometry (Table 3) (Kliphuis et al., 2012). Estimating the maximum specific growth rate directly through growth experiments is complicated. Conditions at which microalgae growth at their maximal growth rate are complicated to maintain in a steady state situation throughout an experiment while measuring biomass concentrations. In addition, growing microalgae at low biomass density with a high incident irradiance destabilizes the cell culture which causes stress responses such as biofilm formation, auto flocculation, and sedimentation. As a result, we chose to monitor the specific oxygen production rate as a proxy for the specific growth rate. Monitoring biological oxygen production is a quick and efficient method estimate microalgal specific growth rates under different culture conditions. *Picochlorum* sp. *(BPE23)* showed the highest specific oxygen production rate out of the three microalgal species, both when grown on urea and on nitrate. *Picochlorum* sp. *(BPE23) reached* this highest specific oxygen production rate at a light intensity of 1500 μmol·m^−2^·s^−1^. The specific oxygen production rate decreased with increasing light intensities above 1500 μmol·m^−2^·s^−1^. For *Nannochloropsis* sp. and *N. oleoabundans* a decrease of the specific oxygen production rate was not observed. This decreasing specific oxygen production rate in *Picochlorum* sp. *(BPE23)* may have been caused by photoinhibition. However, a more likely reason for this decline is the oxygen concentration in the liquid phase which reached values above air saturation in these specific experiments (Supporting Information Appendix 1). These high and oversaturating oxygen concentrations resulted in the formation of gas bubbles which was further stimulated by the rapid mixing of the culture in the biological oxygen monitor. Such gas bubbles serve as a sink for oxygen, leading to a decrease of the dissolved oxygen level in the liquid phase, causing an underestimation of oxygen production rates. For measurements with *Neochloris* and *Nannochloropsis* the oxygen concentration in the liquid phase never surpassed the level of air saturation.

**Table 3:** Values for the estimated maximal specific growth rate ($\mu_{\max}$) based on biological oxygen evolution experiments

Organism	$\mu_{\max}$(day^−1^) (urea)	$\mu_{\max}$ (day^−1^) (nitrate)
*Picochlorum* sp. (BPE23)	4.98 ± 0.24	3.79 ± 0.06
*Nannochloropsis* sp.	2.10 ± 0.19	2.48 ± 0.24
*N. oleoabundans*	2.45 ± 0.05	2.44 ± 0.45

The maximal specific growth rate was then estimated using the highest value of the specific oxygen production rate for each species and growth condition. For *Picochlorum* sp. *(BPE23)* grown on urea a maximum specific growth rate of 4.98 ± 0.24 day^−1^ was calculated, and when grown on nitrate a growth rate of 3.79 ± 0.06 day^−1^ was calculated. *Nannochloropsis* displayed a maximal specific growth rate of 2.10 ± 0.19 day^−1^ with urea as a nitrogen source and 2.48 ± 0.24 day^−1^ with nitrate as nitrogen source. *Neochloris* displayed a maximal specific growth rate of 2.45 ± 0.05 day^−1^ when grown on urea and 2.44 ± 0.45 day^−1^ when grown on nitrate. Comparable maximal specific growth rates have been reported in the literature (de Vree, 2016; Gouveia et al., 2009). Urea and nitrate are both commonly used nitrogen sources for microalgal production. Stoichiometrically maximal specific growth rates using urea should be higher than for nitrate, as nitrate is more reduced than urea and therefore requires a larger investment of energy for conversion to protein (Kliphuis et al., 2012). However, only *Picochlorum* sp. *(BPE23)* grew faster on urea than on nitrate. While in theory urea is energetically favorable, in practice it is unpredictable which nitrogen source yields higher specific maximal growth rates (Arumugam et al., 2013; Wu et al., 2013). Species of *Picochlorum* are known for their high growth rate; Weissman et al. found a growth rate for *Picochlorum Celeri* of 7.9–8.16 day^−1^ (Krishnan et al., 2021; Weissman et al., 2018). Such high growth rates are rarely found in microalgae, as most microalgae show a maximal specific growth rate of 1–3 day^−1^ (Darvehei et al., 2018; Ras et al., 2013; Vree et al., 2015). The specific growth rate of strains within a genus can differ significantly, as seen by the difference in maximal specific growth rate for *P. Celeri* and *Picochlorum sp. (BPE23)*. The variation between species with regard to substrate preference and growth characteristics is why growth parameters have to be determined anew for each strain.

### 3.3 Model simulations

**Figure 3:**
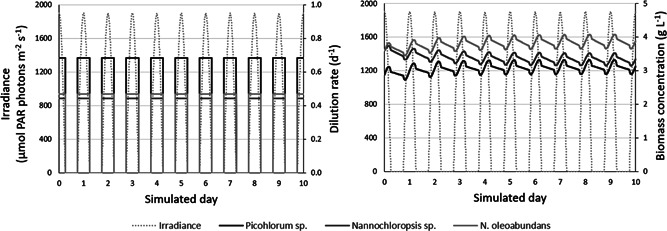
The irradiance levels, dilution rates, and biomass concentrations of the microalgae during the simulated period of 10 identical days

As an example, the biological parameters obtained for *Picochlorum* sp. *(BPE23), Nannochloropsis* sp., and *N. oleoabundans* were used in a microalgal growth model to simulate their potential biomass productivity in a flat panel photobioreactor under chemostat operation conditions (Figure 3). In these simulations, the Caribbean island Bonaire was considered, which is a low‐latitude location with high irradiance throughout the year. Irradiance levels on Day 172 of the year, corresponding to the longest day in the northern hemisphere, were used in the model. Based on generated irradiance data (Supporting Information Appendix 2), assumed operation conditions, obtained biological parameters (Tables 2 and 3), and respective absorption coefficients (Supporting Information Appendix 3), the areal biomass productivity and biomass yield on light were computed for the microalgae. Ten identical days were simulated, during which a pseudo‐steady state is reached (Figure 3). The last day was considered to calculate the biomass productivity of the microalgae.

Our simulations show that *Picochlorum* sp. achieves the highest areal biomass productivities (Table 4); productivities of 32.2 g.m^−2^·day^−1^ were computed, whereas for *N. oleoabundans* and *Nannochloropsis* values of 27.4 and 22.4 g.m^−2^·day^−1^ were found, respectively. Considering the simulated available sunlight on Day 172, namely 52.3 mol_ph_·m^−2^·day^−1^, these productivities correspond to the following photosynthetic efficiencies (PE) normalized to the complete solar spectrum (Supporting Information Appendix 5): 2.8% for *Picochlorum* sp., 2.4% for *N. oleoabundans*, and 2.0% for *Nannochloropsis* sp. (Table 4 and Supporting Information Appendix 5). These values are in a similar range or slightly higher compared with photosynthetic efficiencies reported for outdoor photobioreactor experiments (Ruiz et al., 2016; Vree et al., 2015). For instance, in the Netherlands, average photosynthetic efficiencies of 1.1%–2.4% and areal productivities of 9.7–20.5 g.m^−2^·day^−1^ were achieved with *Nannochloropsis* sp. in outdoor photobioreactors during several weeks in the summer (Vree et al., 2015). It should be noted that studies are difficult to compare directly due to the differences in location, microalgae species, and cultivation conditions.

**Table 4:** Model results for the areal biomass productivity (*r*_*x*,*area*_) and the photosynthetic efficiency (PE) of the microalgae when grown in a photobioreactor on Bonaire

Organism	*r*_*x*,*area*_ (g m^−2^ day^−1^)	PE (%)
*Picochlorum* sp. (BPE23)	32.2	2.8
*Nannochloropsis* sp.	27.4	2.4
*Neochloris oleoabundans*	22.4	2.0
*Picochlorum celeri*	40.6	3.5

*Note*: For the simulations of *Picochlorum* sp. (*BPE23*), *Nannochloropsis* sp., and *Neochloris oleoabundans*, the obtained biological parameters in this study (Tables 2 and 3) were used, whereas for *Picochlorum celeri* a hypothetical simulation was done using its *µ_max_* of 7.9 day^‐1^ (Weissman et al., 2018) in combination with the biological parameters of *Picochlorum* sp. (*BPE23*)

The model results illustrate that, in a photobioreactor at a low‐latitude location such as Bonaire, *Picochlorum* sp. is able to achieve the highest biomass productivities, followed by *N. oleoabundans* and *Nannochloropsis* sp. The differences between the microalgae are largely a result of their maximum specific growth rate; in this study the maximum specific growth rate for *Picochlorum* sp. was found to be more than double that of *Nannochloropsis* sp. and *N. oleoabundans* when grown on urea (Table 3). Other studies show even higher values for *Picochlorum* species. For example, Krishnan et al. (2021) found an exceptional maximum specific growth rate of 7.9 day^−1^ for *Picochlorum celeri* when grown at a relatively high temperature of 33°C and constant light of 900 μmol·m^−2^·s^−1^ in a salt water medium (Krishnan et al., 2021). To discern the effect of such a high maximum growth rate on the final biomass productivity, simulations were performed with the microalgal growth model in which a maximal growth rate of 7.9 day^−1^ was considered in combination with the other measured growth parameters of *Picochlorum sp*. These simulations resulted in high biomass productivities of 40.6 g.m^−2^·day^−1^ at a location such as Bonaire (Table 4). This productivity corresponds to a photosynthetic efficiency of 3.5% (Table 4). The results illustrate the potential of fast‐growing microalgae such as *Picochlorum* to improve biomass productivities and photosynthetic efficiencies.

Our model allows estimation of potential biomass productivities that can be reached based on the determined biological parameters of the microalgae and the irradiance conditions at a low‐latitude location. To model and compare reliable estimates of biomass productivity of different algal species under solar conditions, it is essential to accurately determine the biological parameters of each microalgae strain, including the maximum growth rate, maximum biomass yield on light, and maintenance rate, as was done in this study. These parameters can ultimately be used in different models to study and optimize microalgae production.

## 4 CONCLUDING REMARKS

The growth parameters biomass yield on light, specific maintenance rate and maximal specific growth rate were measured for the microalgae *Picochlorum* sp., *N. oleoabundans*, and *Nannochloropsis* sp. using urea and nitrate as nitrogen sources. *Picochlorum* sp. exhibited the highest maximal specific growth rate with 4.98 ± 0.24 day^−1^, and the lowest specific maintenance rate of 0.079 day^−1^. *N. oleoabundans* displayed the highest yield on light of 1.78 g_x_·mol_ph_^−1^.

A simple growth model was applied to compare biomass productivities at a high‐irradiance location. Based on these model simulations, *Picochlorum* sp. was found to achieve the highest biomass productivity, followed by *N. oleoabundans* and *Nannochloropsis* sp. The measured growth parameters are of significant relevance as they can be applied in more extensive models without further modification, can help to compare microalgal species, and help to manage expectations on the productivities of microalgae cultivation.

## CONFLICT OF INTERESTS

The authors declare that there are no conflict of interests.

## AUTHOR CONTRIBUTIONS

**Robin Barten:** Conceptualization, Data acquisition, Data analysis, Methodology, Writing – original draft. **Rocca Chin‐On**: Data acquisition, Data analysis, Writing – original draft. **Jeroen de Vree:** Methodology, Data acquisition, Data analysis, Writing – review & editing. **Ellen van Beersum:** Methodology, Data acquisition, Data analysis, Writing – review & editing. **Rene H. Wijffels:** Funding acquisition, Writing – review & editing. **Maria J. Barbosa:** Methodology, Funding acquisition, Writing – review & editing. **Marcel Janssen:** Methodology, Writing – review & editing.

## Supporting information

Supporting information.

## Data Availability

The data that support the findings of this study are available from the corresponding author upon reasonable request.
