# Supplementary material for: Growth parameter estimation and model simulation for three industrially relevant microalgae: Picochlorum, Nannochloropsis, and Neochloris
Source: Biotechnol Bioeng. 2022 Feb 19;119(6):1416–25. doi: 10.1002/bit.28052 (PMC9303635; doi:10.1002/bit.28052)
Supplement: Supplementary file 1 — Supporting information. [file BIT-119-1416-s001.docx]

# Appendix 1

Effect of oxygen concentration in the liquid phase on the growth rate of *Picochlorum sp. (BPE23)*. Experiments were performed by monitoring of biological oxygen production (2.1.3. Biological oxygen monitor). The decrease in growth rate was calculated as the difference between the maximal specific growth rate and the growth rate which was measured at the end of the experiment.

Fig A1: The effect of the oxygen concentration on the relative oxygen production rate of Picochlorum sp. (BPE23) during oxygen evolution experiments.

# Appendix 2

Irradiance levels on Bonaire during day 172. Values in W.m^-2^ were obtained from Meteonorm 7.1, based on the measured irradiance at the nearby weather station on Curacao over recent decades. Those values were converted to values in J-PAR.m^-2^.s^-1^ and µmol_-ph_m^-2^.s^-1^ with conversion factors of 0.425 J-PAR.J-sunlight^-1^ and 4.6 µmol_-ph_.J-PAR^-1^.

*Table A1. Irradiance data from Meteonorm 7.1.*

| ***Hour*** | ***Light intensity*** | | |
| --- | --- | --- | --- |
| [h] | [W.m^-2^ =  J-sunlight.s^-1^.m^-2^] | J-PAR.m^-2^.s^-1^ | [µmol_-ph_.m^-2^.s^-1^] |
| 6 | 0 | 0 | 0 |
| 7 | 13 | 6 | 25 |
| 8 | 210 | 89 | 411 |
| 9 | 439 | 187 | 859 |
| 10 | 647 | 275 | 1266 |
| 11 | 822 | 349 | 1608 |
| 12 | 928 | 394 | 1816 |
| 13 | 971 | 413 | 1900 |
| 14 | 945 | 402 | 1849 |
| 15 | 849 | 361 | 1661 |
| 16 | 688 | 292 | 1346 |
| 17 | 478 | 203 | 935 |
| 18 | 252 | 107 | 493 |
| 19 | 27 | 11 | 53 |
| 20 | 0 | 0 | 0 |

# Appendix 3

The absorption cross section was measured for Picochlorum sp. (BPE23), and obtained from literature for Nannochloropsis sp., and Neochloris oleoabundans and used for the model simulations.

*Table A2. Absorption spectra for Picochlorum sp. (BPE23)* for *Nannochloropsis sp.*, and *Neochloris oleoabundans*.

|  | ***Picochlorum sp. (BPE23)*** | ***Nannochloropsis sp.*** | ***Neochloris oleoabundans*** |
| --- | --- | --- | --- |
| Reference: | This study | Benvenuti et al. 2014 | Klok et al. 2013 |
| Wavelength [nm] | Absorption [m^2^.mol^-1^] | Absorption [m^2^.mol^-1^] | Absorption [m^2^.mol^-1^] |
| 400 | 7.7 | 6.2 | 5.8 |
| 401 | 7.7 | 6.5 | 5.8 |
| 402 | 7.9 | 6.5 | 5.9 |
| 403 | 7.9 | 6.5 | 6.1 |
| 404 | 8.1 | 6.5 | 6.2 |
| 405 | 8.2 | 6.8 | 6.3 |
| 406 | 8.3 | 6.9 | 6.4 |
| 407 | 8.4 | 7.0 | 6.5 |
| 408 | 8.6 | 7.0 | 6.6 |
| 409 | 8.7 | 7.1 | 6.7 |
| 410 | 8.8 | 7.3 | 6.8 |
| 411 | 9.0 | 7.5 | 6.9 |
| 412 | 9.1 | 7.5 | 7.0 |
| 413 | 9.2 | 7.6 | 7.1 |
| 414 | 9.2 | 7.6 | 7.2 |
| 415 | 9.3 | 7.6 | 7.3 |
| 416 | 9.5 | 7.7 | 7.3 |
| 417 | 9.3 | 7.8 | 7.4 |
| 418 | 9.5 | 7.9 | 7.5 |
| 419 | 9.5 | 8.0 | 7.5 |
| 420 | 9.7 | 8.0 | 7.6 |
| 421 | 9.6 | 8.0 | 7.6 |
| 422 | 9.7 | 8.0 | 7.7 |
| 423 | 9.7 | 8.0 | 7.7 |
| 424 | 9.9 | 8.2 | 7.8 |
| 425 | 9.9 | 8.1 | 7.8 |
| 426 | 10.0 | 8.2 | 7.9 |
| 427 | 10.0 | 8.4 | 8.0 |
| 428 | 10.1 | 8.4 | 8.0 |
| 429 | 10.1 | 8.4 | 8.1 |
| 430 | 10.2 | 8.4 | 8.2 |
| 431 | 10.4 | 8.6 | 8.3 |
| 432 | 10.4 | 8.5 | 8.4 |
| 433 | 10.4 | 8.8 | 8.4 |
| 434 | 10.5 | 8.7 | 8.5 |
| 435 | 10.5 | 8.8 | 8.5 |
| 436 | 10.5 | 8.8 | 8.5 |
| 437 | 10.6 | 8.8 | 8.5 |
| 438 | 10.5 | 8.7 | 8.5 |
| 439 | 10.5 | 8.7 | 8.5 |
| 440 | 10.4 | 8.7 | 8.5 |
| 441 | 10.4 | 8.7 | 8.4 |
| 442 | 10.2 | 8.6 | 8.3 |
| 443 | 10.1 | 8.4 | 8.2 |
| 444 | 10.0 | 8.4 | 8.1 |
| 445 | 9.9 | 8.3 | 8.0 |
| 446 | 9.6 | 8.0 | 7.8 |
| 447 | 9.5 | 7.8 | 7.7 |
| 448 | 9.2 | 7.7 | 7.5 |
| 449 | 9.1 | 7.5 | 7.4 |
| 450 | 9.0 | 7.4 | 7.2 |
| 451 | 8.7 | 7.2 | 7.1 |
| 452 | 8.6 | 7.0 | 6.9 |
| 453 | 8.4 | 7.0 | 6.8 |
| 454 | 8.3 | 6.7 | 6.7 |
| 455 | 8.3 | 6.5 | 6.6 |
| 456 | 8.3 | 6.5 | 6.6 |
| 457 | 8.2 | 6.4 | 6.5 |
| 458 | 8.2 | 6.3 | 6.5 |
| 459 | 8.1 | 6.3 | 6.4 |
| 460 | 8.1 | 6.2 | 6.4 |
| 461 | 8.1 | 6.2 | 6.4 |
| 462 | 8.1 | 6.1 | 6.3 |
| 463 | 8.1 | 6.0 | 6.3 |
| 464 | 8.2 | 6.0 | 6.3 |
| 465 | 8.2 | 5.9 | 6.3 |
| 466 | 8.2 | 5.8 | 6.3 |
| 467 | 8.2 | 5.7 | 6.3 |
| 468 | 8.2 | 5.8 | 6.3 |
| 469 | 8.1 | 5.6 | 6.3 |
| 470 | 8.2 | 5.6 | 6.3 |
| 471 | 8.2 | 5.5 | 6.3 |
| 472 | 8.2 | 5.5 | 6.3 |
| 473 | 8.2 | 5.6 | 6.3 |
| 474 | 8.2 | 5.5 | 6.3 |
| 475 | 8.2 | 5.5 | 6.3 |
| 476 | 8.2 | 5.5 | 6.3 |
| 477 | 8.2 | 5.5 | 6.3 |
| 478 | 8.2 | 5.5 | 6.3 |
| 479 | 8.2 | 5.5 | 6.2 |
| 480 | 8.2 | 5.6 | 6.2 |
| 481 | 8.1 | 5.6 | 6.2 |
| 482 | 8.1 | 5.6 | 6.2 |
| 483 | 8.1 | 5.7 | 6.1 |
| 484 | 8.1 | 5.7 | 6.1 |
| 485 | 7.9 | 5.7 | 6.1 |
| 486 | 7.8 | 5.7 | 6.0 |
| 487 | 7.8 | 5.7 | 6.0 |
| 488 | 7.7 | 5.6 | 5.9 |
| 489 | 7.6 | 5.7 | 5.8 |
| 490 | 7.3 | 5.6 | 5.7 |
| 491 | 7.2 | 5.5 | 5.6 |
| 492 | 7.0 | 5.5 | 5.5 |
| 493 | 6.9 | 5.4 | 5.4 |
| 494 | 6.7 | 5.4 | 5.3 |
| 495 | 6.5 | 5.3 | 5.1 |
| 496 | 6.4 | 5.3 | 5.0 |
| 497 | 6.1 | 5.2 | 4.9 |
| 498 | 6.0 | 5.1 | 4.7 |
| 499 | 5.8 | 5.0 | 4.5 |
| 500 | 5.6 | 4.9 | 4.4 |
| 501 | 5.4 | 4.8 | 4.2 |
| 502 | 5.1 | 4.7 | 4.0 |
| 503 | 4.9 | 4.6 | 3.9 |
| 504 | 4.7 | 4.4 | 3.7 |
| 505 | 4.5 | 4.3 | 3.5 |
| 506 | 4.4 | 4.2 | 3.4 |
| 507 | 4.1 | 4.1 | 3.2 |
| 508 | 4.0 | 3.9 | 3.0 |
| 509 | 3.7 | 3.8 | 2.9 |
| 510 | 3.6 | 3.7 | 2.7 |
| 511 | 3.5 | 3.5 | 2.6 |
| 512 | 3.2 | 3.4 | 2.5 |
| 513 | 3.1 | 3.4 | 2.3 |
| 514 | 2.9 | 3.1 | 2.2 |
| 515 | 2.7 | 3.0 | 2.1 |
| 516 | 2.6 | 2.9 | 2.0 |
| 517 | 2.4 | 2.8 | 1.9 |
| 518 | 2.2 | 2.7 | 1.8 |
| 519 | 2.2 | 2.5 | 1.7 |
| 520 | 2.0 | 2.5 | 1.6 |
| 521 | 1.9 | 2.3 | 1.5 |
| 522 | 1.9 | 2.2 | 1.5 |
| 523 | 1.8 | 2.1 | 1.4 |
| 524 | 1.8 | 2.1 | 1.3 |
| 525 | 1.7 | 1.9 | 1.3 |
| 526 | 1.7 | 1.9 | 1.2 |
| 527 | 1.5 | 1.8 | 1.2 |
| 528 | 1.5 | 1.8 | 1.2 |
| 529 | 1.5 | 1.7 | 1.1 |
| 530 | 1.4 | 1.6 | 1.1 |
| 531 | 1.4 | 1.6 | 1.1 |
| 532 | 1.4 | 1.6 | 1.0 |
| 533 | 1.4 | 1.5 | 1.0 |
| 534 | 1.3 | 1.4 | 1.0 |
| 535 | 1.3 | 1.4 | 1.0 |
| 536 | 1.3 | 1.3 | 1.0 |
| 537 | 1.4 | 1.3 | 1.0 |
| 538 | 1.3 | 1.2 | 0.9 |
| 539 | 1.3 | 1.2 | 0.9 |
| 540 | 1.3 | 1.2 | 0.9 |
| 541 | 1.3 | 1.2 | 0.9 |
| 542 | 1.3 | 1.1 | 0.9 |
| 543 | 1.3 | 1.1 | 0.9 |
| 544 | 1.3 | 1.1 | 0.9 |
| 545 | 1.3 | 1.1 | 0.9 |
| 546 | 1.2 | 1.0 | 0.9 |
| 547 | 1.3 | 1.0 | 0.9 |
| 548 | 1.3 | 0.9 | 0.8 |
| 549 | 1.3 | 0.9 | 0.8 |
| 550 | 1.3 | 0.9 | 0.8 |
| 551 | 1.3 | 0.9 | 0.8 |
| 552 | 1.3 | 0.9 | 0.8 |
| 553 | 1.2 | 0.8 | 0.8 |
| 554 | 1.2 | 0.9 | 0.8 |
| 555 | 1.3 | 0.8 | 0.8 |
| 556 | 1.3 | 0.8 | 0.8 |
| 557 | 1.3 | 0.8 | 0.8 |
| 558 | 1.3 | 0.8 | 0.8 |
| 559 | 1.3 | 0.8 | 0.8 |
| 560 | 1.3 | 0.9 | 0.8 |
| 561 | 1.3 | 0.8 | 0.8 |
| 562 | 1.3 | 0.8 | 0.8 |
| 563 | 1.3 | 0.8 | 0.9 |
| 564 | 1.4 | 0.8 | 0.9 |
| 565 | 1.4 | 0.8 | 0.9 |
| 566 | 1.4 | 0.9 | 0.9 |
| 567 | 1.4 | 0.8 | 0.9 |
| 568 | 1.4 | 0.9 | 1.0 |
| 569 | 1.5 | 0.9 | 1.0 |
| 570 | 1.5 | 0.9 | 1.0 |
| 571 | 1.5 | 0.9 | 1.0 |
| 572 | 1.5 | 0.9 | 1.0 |
| 573 | 1.7 | 0.9 | 1.1 |
| 574 | 1.7 | 1.0 | 1.1 |
| 575 | 1.8 | 1.0 | 1.1 |
| 576 | 1.8 | 1.0 | 1.1 |
| 577 | 1.8 | 1.0 | 1.2 |
| 578 | 1.8 | 1.0 | 1.2 |
| 579 | 1.8 | 1.0 | 1.2 |
| 580 | 1.9 | 1.1 | 1.2 |
| 581 | 1.9 | 1.1 | 1.2 |
| 582 | 1.9 | 1.1 | 1.3 |
| 583 | 1.9 | 1.1 | 1.3 |
| 584 | 1.9 | 1.1 | 1.3 |
| 585 | 2.0 | 1.1 | 1.3 |
| 586 | 2.0 | 1.2 | 1.3 |
| 587 | 2.0 | 1.2 | 1.4 |
| 588 | 2.0 | 1.2 | 1.4 |
| 589 | 2.0 | 1.2 | 1.4 |
| 590 | 2.0 | 1.2 | 1.4 |
| 591 | 2.2 | 1.2 | 1.4 |
| 592 | 2.2 | 1.2 | 1.5 |
| 593 | 2.2 | 1.2 | 1.5 |
| 594 | 2.2 | 1.2 | 1.5 |
| 595 | 2.2 | 1.2 | 1.5 |
| 596 | 2.2 | 1.2 | 1.5 |
| 597 | 2.2 | 1.2 | 1.5 |
| 598 | 2.2 | 1.2 | 1.5 |
| 599 | 2.2 | 1.3 | 1.6 |
| 600 | 2.3 | 1.2 | 1.6 |
| 601 | 2.3 | 1.3 | 1.6 |
| 602 | 2.3 | 1.3 | 1.6 |
| 603 | 2.3 | 1.3 | 1.6 |
| 604 | 2.3 | 1.3 | 1.7 |
| 605 | 2.3 | 1.4 | 1.7 |
| 606 | 2.3 | 1.5 | 1.7 |
| 607 | 2.3 | 1.4 | 1.7 |
| 608 | 2.4 | 1.5 | 1.8 |
| 609 | 2.4 | 1.6 | 1.8 |
| 610 | 2.4 | 1.6 | 1.8 |
| 611 | 2.4 | 1.6 | 1.8 |
| 612 | 2.6 | 1.7 | 1.9 |
| 613 | 2.6 | 1.7 | 1.9 |
| 614 | 2.7 | 1.8 | 1.9 |
| 615 | 2.7 | 1.8 | 2.0 |
| 616 | 2.7 | 1.9 | 2.0 |
| 617 | 2.7 | 1.9 | 2.0 |
| 618 | 2.8 | 1.9 | 2.0 |
| 619 | 2.8 | 2.0 | 2.1 |
| 620 | 2.8 | 2.0 | 2.1 |
| 621 | 2.8 | 2.0 | 2.1 |
| 622 | 2.8 | 2.1 | 2.1 |
| 623 | 2.8 | 2.1 | 2.1 |
| 624 | 2.8 | 2.1 | 2.1 |
| 625 | 2.8 | 2.1 | 2.1 |
| 626 | 2.8 | 2.2 | 2.2 |
| 627 | 2.9 | 2.1 | 2.2 |
| 628 | 2.9 | 2.2 | 2.2 |
| 629 | 2.9 | 2.2 | 2.2 |
| 630 | 2.8 | 2.1 | 2.2 |
| 631 | 2.8 | 2.1 | 2.2 |
| 632 | 2.9 | 2.1 | 2.2 |
| 633 | 2.8 | 2.1 | 2.2 |
| 634 | 2.9 | 2.1 | 2.2 |
| 635 | 2.9 | 2.1 | 2.2 |
| 636 | 2.9 | 2.0 | 2.2 |
| 637 | 3.1 | 2.0 | 2.2 |
| 638 | 3.1 | 2.0 | 2.3 |
| 639 | 3.1 | 1.9 | 2.3 |
| 640 | 3.3 | 1.9 | 2.3 |
| 641 | 3.3 | 1.9 | 2.4 |
| 642 | 3.5 | 1.8 | 2.4 |
| 643 | 3.7 | 1.8 | 2.5 |
| 644 | 3.8 | 1.8 | 2.6 |
| 645 | 4.0 | 1.8 | 2.7 |
| 646 | 4.2 | 1.7 | 2.8 |
| 647 | 4.4 | 1.8 | 2.9 |
| 648 | 4.5 | 1.8 | 3.0 |
| 649 | 4.6 | 1.9 | 3.1 |
| 650 | 4.6 | 1.9 | 3.2 |
| 651 | 4.7 | 2.0 | 3.3 |
| 652 | 4.7 | 2.0 | 3.4 |
| 653 | 4.7 | 2.2 | 3.4 |
| 654 | 4.7 | 2.3 | 3.5 |
| 655 | 4.7 | 2.4 | 3.6 |
| 656 | 4.7 | 2.6 | 3.7 |
| 657 | 4.9 | 2.8 | 3.8 |
| 658 | 5.0 | 3.0 | 3.9 |
| 659 | 5.1 | 3.3 | 4.0 |
| 660 | 5.2 | 3.5 | 4.2 |
| 661 | 5.5 | 3.7 | 4.3 |
| 662 | 5.8 | 4.0 | 4.5 |
| 663 | 5.9 | 4.3 | 4.7 |
| 664 | 6.1 | 4.5 | 4.9 |
| 665 | 6.3 | 4.8 | 5.1 |
| 666 | 6.7 | 5.0 | 5.3 |
| 667 | 6.8 | 5.3 | 5.5 |
| 668 | 7.0 | 5.5 | 5.6 |
| 669 | 7.2 | 5.7 | 5.8 |
| 670 | 7.4 | 5.9 | 5.9 |
| 671 | 7.6 | 6.2 | 6.0 |
| 672 | 7.7 | 6.4 | 6.1 |
| 673 | 7.8 | 6.5 | 6.2 |
| 674 | 7.9 | 6.7 | 6.3 |
| 675 | 7.9 | 6.8 | 6.3 |
| 676 | 7.9 | 6.9 | 6.3 |
| 677 | 7.9 | 7.0 | 6.3 |
| 678 | 8.1 | 7.0 | 6.3 |
| 679 | 7.9 | 7.0 | 6.3 |
| 680 | 8.1 | 7.0 | 6.3 |
| 681 | 7.9 | 6.9 | 6.2 |
| 682 | 7.8 | 6.7 | 6.1 |
| 683 | 7.4 | 6.6 | 5.9 |
| 684 | 7.2 | 6.3 | 5.7 |
| 685 | 6.8 | 6.0 | 5.5 |
| 686 | 6.3 | 5.7 | 5.2 |
| 687 | 5.8 | 5.3 | 4.8 |
| 688 | 5.1 | 4.9 | 4.5 |
| 689 | 4.6 | 4.5 | 4.1 |
| 690 | 4.1 | 4.1 | 3.8 |
| 691 | 3.6 | 3.6 | 3.4 |
| 692 | 3.2 | 3.3 | 3.1 |
| 693 | 2.7 | 2.9 | 2.8 |
| 694 | 2.3 | 2.5 | 2.5 |
| 695 | 2.0 | 2.2 | 2.2 |
| 696 | 1.8 | 1.9 | 1.9 |
| 697 | 1.5 | 1.7 | 1.7 |
| 698 | 1.4 | 1.5 | 1.5 |
| 699 | 1.3 | 1.3 | 1.3 |
| 700 | 1.2 | 1.1 | 1.2 |

# Appendix 4

Simulation results for each microalgae to determine the optimal dilution rate from the calculated biomass productivity and yield on light.

*Table A3. Model simulation results*

| ***Picochlorum sp. (BPE23)*** | | | | | | |
| --- | --- | --- | --- | --- | --- | --- |
| **Cx0** | | **Dilution rate** | | **Biomass productivity** | | **Biomass yield on light** |
| [mol.m^-3^] | [g.L^-1^] | [s^-1^] | [d^-1^] | [g.L^-1^.d^-1^] | [g.m^-2^.d^-1^] | [g_x_.mol_ph_^-1^] |
| 120 | 2.9 | 1.67E-05 | 0.72 | 2.146 | 32.2 | 0.616 |
| 120 | 2.9 | 1.39E-05 | 0.60 | 2.140 | 32.1 | 0.614 |
| 120 | 2.9 | 1.94E-05 | 0.84 | 2.129 | 31.9 | 0.611 |
| 120 | 2.9 | 1.53E-05 | 0.66 | 2.146 | 32.2 | 0.616 |
| 120 | 2.9 | 1.58E-05 | 0.68 | 2.147 | 32.2 | 0.616 |
| 120 | 2.9 | 1.61E-05 | 0.70 | 2.147 | 32.2 | 0.616 |
| 120 | 2.9 | 1.64E-05 | 0.71 | 2.146 | 32.2 | 0.616 |
| 120 | 2.9 | 1.56E-05 | 0.67 | 2.147 | 32.2 | 0.616 |
| 120 | 2.9 | 1.11E-05 | 0.48 | 2.105 | 31.6 | 0.604 |
| 120 | 2.9 | 2.22E-05 | 0.96 | 2.095 | 31.4 | 0.601 |

| ***Neochloris oleoabundans*** | | | | | | |
| --- | --- | --- | --- | --- | --- | --- |
| **Cx0** | | **Dilution rate** | | **Biomass productivity** | | **Biomass yield on light** |
| [mol.m^-3^] | [g.L^-1^] | [s^-1^] | [d^-1^] | [g.L^-1^.d^-1^] | [g.m^-2^.d^-1^] | [g_x_.mol_ph_^-1^] |
| 100 | 2.4 | 1.67E-05 | 0.72 | 1.676 | 25.1 | 0.481 |
| 120 | 2.9 | 1.39E-05 | 0.60 | 1.782 | 26.7 | 0.512 |
| 100 | 2.4 | 1.94E-05 | 0.84 | 1.505 | 22.6 | 0.432 |
| 120 | 2.9 | 1.11E-05 | 0.48 | 1.826 | 27.4 | 0.524 |
| 150 | 3.6 | 8.33E-06 | 0.36 | 1.799 | 27.0 | 0.516 |
| 150 | 3.6 | 9.72E-06 | 0.42 | 1.822 | 27.3 | 0.523 |
| 150 | 3.6 | 1.06E-05 | 0.46 | 1.827 | 27.4 | 0.524 |
| 150 | 3.6 | 1.08E-05 | 0.47 | 1.827 | 27.4 | 0.524 |
| 150 | 3.6 | 1.03E-05 | 0.44 | 1.826 | 27.4 | 0.524 |

| ***Nannochloropsis sp.*** | | | | | | |
| --- | --- | --- | --- | --- | --- | --- |
| **Cx0** | | **Dilution rate** | | **Biomass productivity** | | **Biomass yield on light** |
| [mol.m^-3^] | [g.L^-1^] | [s^-1^] | [d^-1^] | [g.L^-1^.d^-1^] | [g.m^-2^.d^-1^] | [g_x_.mol_ph_^-1^] |
| 100 | 2.4 | 1.67E-05 | 0.72 | 1.335 | 20.0 | 0.383 |
| 100 | 2.4 | 1.39E-05 | 0.60 | 1.437 | 21.6 | 0.412 |
| 100 | 2.4 | 1.94E-05 | 0.84 | 1.182 | 17.7 | 0.339 |
| 70 | 1.7 | 1.94E-05 | 0.84 | 1.178 | 17.7 | 0.338 |
| 100 | 2.4 | 1.11E-05 | 0.48 | 1.487 | 22.3 | 0.427 |
| 150 | 3.6 | 8.33E-06 | 0.36 | 1.476 | 22.1 | 0.424 |
| 150 | 3.6 | 9.72E-06 | 0.42 | 1.491 | 22.4 | 0.428 |
| 150 | 3.6 | 1.03E-05 | 0.44 | 1.491 | 22.4 | 0.428 |
| 150 | 3.6 | 1.00E-05 | 0.43 | 1.491 | 22.4 | 0.428 |
| 150 | 3.6 | 1.06E-05 | 0.46 | 1.491 | 22.4 | 0.428 |
| 150 | 3.6 | 1.08E-05 | 0.47 | 1.49 | 22.4 | 0.428 |

| ***Picochlorum celeri*** | | | | | | |
| --- | --- | --- | --- | --- | --- | --- |
| **Cx0** | | **Dilution rate** | | **Biomass productivity** | | **Biomass yield on light** |
| [mol.m^-3^] | [g.L^-1^] | [s^-1^] | [d^-1^] | [g.L^-1^.d^-1^] | [g.m^-2^.d^-1^] | [g_x_.mol_ph_^-1^] |
| 120 | 2.9 | 2.22E-05 | 0.96 | 2.700 | 40.5 | 0.775 |
| 120 | 2.9 | 2.50E-05 | 1.08 | 2.683 | 40.2 | 0.770 |
| 120 | 2.9 | 1.94E-05 | 0.84 | 2.704 | 40.6 | 0.776 |
| 120 | 2.9 | 1.67E-05 | 0.72 | 2.692 | 40.4 | 0.773 |
| 120 | 2.9 | 2.08E-05 | 0.90 | 2.704 | 40.6 | 0.776 |
| 120 | 2.9 | 2.03E-05 | 0.88 | 2.704 | 40.6 | 0.776 |
| 120 | 2.9 | 2.00E-05 | 0.86 | 2.704 | 40.6 | 0.776 |
| 120 | 2.9 | 2.14E-05 | 0.92 | 2.703 | 40.5 | 0.776 |

# Appendix 5

The photosynthetic efficiencies were determined by first calculating the available amount of light energy from the available photons. This light energy was calculated with a conversion factor of 4.6 µmol PAR photons / J PAR photons, which resulted in a value of 1.1 ∙ 10^7^ J PAR photons m^-2^ d^-1^. Then, the energy content of the produced biomass was calculated. For the latter, the following conversion factors were used: 24 g.mol_x_^-1^ and 5.59 ∙ 10^5^ J.mol-biomass^-1^ (Janssen, 2016). The photosynthetic efficiencies could then be calculated as the fraction of the energy content of the produced biomass over the available light energy. A distinction is made between the light energy in the PAR-range and the complete sunlight spectrum. It was assumed that the PAR-fraction constitutes to 42.5% of the complete sunlight spectrum.

*Table A4. The calculated biomass productivity and photosynthetic efficiency of the microalgal cultures in a photobioreactor on Bonaire.*

|  | **Biomass productivity and energy content** | | | **Photosynthetic efficiency** | | |
| --- | --- | --- | --- | --- | --- | --- |
|  | [g.m^-2^.d^-1^] | [mol.m^-2^d^-1^] | [J.m^-2^d^-1^] | PAR-range (%) | Sunlight  (%) | [g- biomass.mol-ph^-1^] |
| *Picochlorum sp.* | 32 | 1.3 | 7.5 ∙ 10^5^ | 6.6 | 2.8 | 0.62 |
| *N. oleoabundans* | 27 | 1.1 | 6.4 ∙ 10^5^ | 5.6 | 2.4 | 0.52 |
| *Nannochloropsis sp.* | 22 | 0.93 | 5.2 ∙ 10^5^ | 4.6 | 2.0 | 0.43 |
| *Picochlorum celeri* | 41 | 1.7 | 9.4 ∙ 10^5^ | 8.3 | 3.5 | 0.78 |

# Appendix 6

*Table A5: The biological oxygen monitor experiment was performed using the following light conditions*

| Light level (u_molph_.m^-2^.s^-1^) | Time interval (minutes) |
| --- | --- |
| 0 | 6 |
| 10 | 4 |
| 20 | 3 |
| 40 | 2 |
| 80 | 2 |
| 160 | 1 |
| 250 | 1 |
| 350 | 1 |
| 500 | 1 |
| 750 | 1 |
| 1000 | 1 |
| 1500 | 1 |
| 2000 | 1 |
| 2500 | 1 |
